# Status of evolutionary medicine within the field of nutrition and dietetics: A survey of professionals and students

**DOI:** 10.1093/emph/eoy022

**Published:** 2018-08-08

**Authors:** Anthony J Basile, David B Schwartz, Joseph Rigdon, Hamilton Stapell

**Affiliations:** 1 School of Life Sciences, Arizona State University, 427 E Tyler Mall #4601, Tempe, AZ, USA; 2 Institute of Human Nutrition, Columbia University Medical Center, 630 West 168th Street PH1512, New York, NY, USA; 3 Department of Sociology, Princeton University260 College Ave Apt. E, Palo Alto, CA, USA; 4 Quantitative Science Unit, Stanford University School of Medicine, 1070 Arastradero Rd, Palo Alto, Stanford, CA, USA; 5 Department of History, State University of New York, 600 Hawk Dr, New Paltz, NY, USA

**Keywords:** nutrition, dietetics, evolutionary medicine, interdisciplinary approaches

## Abstract

**Lay Summary:**

Through an online survey of nutrition and dietetic professionals and students, we learned there is interest to incorporate evolutionary medicine into the nutrition and dietetics field and education programs.

**Background and objectives:**

Evolutionary medicine is an emerging field that examines the evolutionary significance of modern disease to develop new preventative strategies or treatments. While many areas of interest in evolutionary medicine and public health involve diet, we currently lack an understanding of whether nutrition and dietetics professionals and students appreciate the potential of evolutionary medicine.

**Methodology:**

Cross-sectional online survey to measure the level of appreciation, applicability and knowledge of evolutionary medicine among nutrition and dietetics professionals and students. We then examined the relationships between support of evolutionary medicine and (i) professionals and students, (ii) US region, (iii) religious belief and (iv) existing evolutionary knowledge.

**Results:**

A total of 2039 people participated: students (*n* = 893) and professionals (*n* = 1146). The majority of the participants agree they are knowledgeable on the theory of evolution (59%), an understanding of evolution can aid the nutrition and dietetics field (58%), an evolutionary perspective would be beneficial in dietetics education (51%) and it is equally important to understand both the evolutionary and direct causes of disease (71%). Significant differences in responses between professionals and students suggest students are currently learning more about evolution and are also more supportive of using an evolutionary perspective. Whereas differences in responses by US region were minimal, differences by religious belief and prior evolutionary knowledge were significant; however, all responses were either neutral or supportive at varying strengths.

**Conclusion and implications:**

There is interest among professionals and students to incorporate evolutionary medicine into the nutrition and dietetics field and education programs.

## INTRODUCTION

Evolutionary medicine is an emerging field that examines the evolutionary significance of modern disease to develop new preventative strategies or treatments [1–3]. In addition to joining evolutionary biology with medicine, it has been suggested that nutrition science can also benefit from an evolutionary perspective [4]. One key area of evolutionary medicine is evolutionary mismatch, where environmental changes can occur more quickly than genomic changes [1, 2]. Specifically, literature supports the notion that the westernized diet is mismatched to the human genome, which evolved with an ancestral/hunter-gatherer diet, contributing to many ‘diseases of civilization,’ including diabetes, obesity and cardiovascular disease [5, 6].

Today, an estimated 70% of caloric intake from the westernized diet comes from food groups unavailable prior to the agricultural revolution [5]. Due to this dietary mismatch, only 9 and 12% of US adults meet the intake recommendations for vegetables and fruit, respectively [7]. Further, nearly one-third of the US population is at risk of deficiency in at least one vitamin or has iron-deficiency anemia [8]. From nutrient deficiencies to metabolic disease, examining today’s nutrition-related disease from an evolutionary perspective may aid in novel therapeutic approaches and benefit large-scale public health initiatives.

A considerable amount of clinical research has recently examined the health benefits of an evolutionary perspective on diet [9–14]. A 2015 systematic review and meta-analysis found an evolutionary perspective on diet produced greater improvements in metabolic syndrome components compared to guideline-based diets [13]. These studies provide evidence that this interdisciplinary approach to dietary recommendations may be beneficial and should be of interest to the nutrition and dietetics field.

Though many advocate for adding an evolutionary perspective into medical school curricula and into the field itself [15–18], there is a well-reported gap between evolutionary biology and medicine, nursing and public health [19]. Hidaka et al. reported that, although the coverage of evolutionary content in medical school has increased, the perceived importance of evolutionary topics as reported by medical school deans is higher than the level of coverage in curricula [20]. Currently, education on evolution and how it relates to dietary therapy is not required by the Accreditation Council for Education in Nutrition and Dietetics (ACEND) [21–27], suggesting an even larger gap between evolutionary medicine and nutrition and dietetics education.

While many areas of interest in evolutionary medicine and public health involve diet, we currently lack an understanding of whether nutrition and dietetics professionals and students appreciate the potential of evolutionary medicine. We therefore tested several hypotheses: (i) students are not learning about evolution and its application to health and disease, (ii) the field of nutrition and dietetics does not appreciate evolutionary medicine, resulting in low support throughout the survey, (iii) professionals and students will have different views on evolutionary medicine, (iv) since US regions are so distinct, responses will vary by region, (v) if religious beliefs challenge the theory of evolution, the participant will be less supportive of evolutionary medicine and (vi) self-reported evolutionary knowledge is associated with appreciation of evolutionary medicine.

## METHODS

### Study design

We distributed a cross-sectional survey using a self-administered online questionnaire. The study population consisted of adults who identified themselves as dietetic students (undergraduate, graduate or dietetic interns) or professionals (registered dietitian nutritionists (RDN) and nutrition and dietetic technicians, registered (NDTR)) in the US and Puerto Rico. The research team created most survey questions while some questions were drawn from an existing survey [28]. To ensure proper interpretation, the survey questions were pilot tested by four RDNs, two dietetics students (one was a NDTR) and one dietetic intern, and edits were made based on feedback.

Due to the often contentious debate surrounding evolution [29, 30], we titled the survey ‘Outside Influence in the Field of Nutrition and Dietetics’ to not deter those with an aversion to evolution from participating. Additionally, the word ‘interdisciplinary’ was excluded from the title as this word may be misinterpreted. The 26-question survey was identical between the two groups except for minor word adjustments specific to each group.

Survey questions collected information about demographics, religious beliefs, evolutionary education and understanding, evolutionary application and clinical applicability (Supplementary Data S1). We then examined the relationships between support of evolutionary medicine and (i) professionals and students, (ii) US region, (iii) religious belief and (iv) existing evolutionary knowledge. The survey was managed through the online program Qualtrics, an Internet-based management system (qaultrics.com). Inclusion criteria included: access to the survey, being 18 years or older, providing consent and being a nutrition and dietetics professional or student.

### Survey distribution

Dietetic education programs directors listed on the Academy of Nutrition and Dietetics’ (AND) website (eatright.org) were contacted by email. The website yielded email contacts for didactic programs (*n* = 223), coordinated programs (*n* = 56), NDTR programs (*n* = 41) and dietetic internships (*n* = 246), providing 561 unique contacts. Program directors received an email that described the survey, encouraged their participation and provided an email addressed to students for ease of survey forwarding.

Dietetic professionals were contacted through email addresses collected from AND’s website. The website yielded email contacts from state affiliate organizations (*n* = 53), dietetic practice groups (*n* = 36) and the ‘Find an Expert’ page (*n* = 5205). State affiliates and dietetic practice group presidents were asked to share the link-containing email request with their group members, whereas ‘Find an Expert’ professionals were directly emailed requesting their participation.

Student survey distribution began 16 March 2016 and ended 14 April 2016 (30 days). Professional survey distribution began 14 May 2016 and ended 6 July 2016 (53 days—extended due to a steady participation rate). A follow-up email was sent 2 weeks after initial contact. To incentivize participation, participants could opt into a drawing for a $50 Amazon gift card.

### Participants

A total of 2167 participants from both survey groups consented. Participants who consented but did not participate more than defining student/professional status were removed, yielding 893 students and 1146 professionals (*n* = 2039). A response rate was unattainable due to distribution methods, as it is impossible to know how many received the survey. Completion rate was 92% for all participants. The dropout rate, based on the number of participants who never completed the last question, was 14%.

### Use of human subjects

Permission was granted from State University of New York, New Paltz, Human Research Ethics Board for use of human subjects.

### Statistical analysis

Fisher’s exact test was used to test categorical variables across two or more levels. A Pearson chi-squared test was used to compare the distribution of belief in evolution in our survey to the national distribution, as measured by a Gallup survey [28]. Statistical tests were applied using R version 3.4.0 [31]. Due to the volume of hypotheses tested and ensuing problem of multiple testing [32], only *P* < 0.0001 were considered significant. Qualitative data provided by participants were analysed with *NVivo 11.*

## RESULTS

### Demographics

Preliminary results were published as an extended abstract [33]. Student participants represented <4% of the 2015–2016 national nutrition and dietetics student population (*n* = 23 594) [34]. Professional participants represented over 1% of the dietetic professionals in 2016 (*n* = 101 165) [35]. With 94% of participants identifying as female, the sample gender is similar to the field in 2017 [35].

Major demographic information can be found in Table 1. Student participants came from 35 US states and Puerto Rico. Professional participants came from all 50 US states, Washington, D.C., and Puerto Rico. Participant distribution was similar between the four US census regions (Northeast 26%, South 26%, Midwest 25% and West 23%). There was approximately a 19-year difference between professionals and students.

**Table 1.** Participant demographics

Students (*n* = 893)			Professionals (*n* = 1146)		
Age	Mean	SD	Age	Mean	SD
	25	6.3		44	13.1
Gender (*n* = 785)			Gender (*n* = 986)		
Female	92%		Female	96%	
Male	8%		Male	4%	
Educational program (*n* = 893)			Professional title (*n* = 1146)		
Undergraduate	53%		Registered dietitian nutritionist	98%	
Graduate	20%				
			Dietetic technician, registered	2%	
Dietetic intern	28%				
Regional location (*n* = 758)			Regional location (*n* = 966)		
Northeast	23%		Northeast	28%	
South	23%		South	28%	
Midwest	30%		Midwest	21%	
West	24%		West	23%	

Roughly 77% (*n* = 1420) of participants believed ‘humans developed over millions of years from less advanced forms of life.’ Of total participants, 45% believed in God-guided evolution, 33% believed in evolution without guidance by God and 23% of participants believed ‘God created human beings pretty much in their present form at one time within the last 10 000 years or so’. Our results are significantly different from participant responses to the 2018 Gallup poll [28] (*X*^2^ = 401.68, df = 3, *P*-value < 2.2e-16). To test whether participant dropouts had an aversion to the theory of evolution, we classified all dropouts (*n* = 197) as those who believe God made humans 10 000 years ago. The results remain significantly different from the 2018 Gallup data (*X*^2^ = 252.58, df = 3, *P*-value < 2.2e-16).

Approximately 49% of participants believed Charles Darwin’s theory of evolution is a scientific theory well-supported by evidence, while 28% believed Charles Darwin’s theory of evolution is just one of many scientific theories and is not well-supported by evidence, and 23% reported they did not know enough to have an opinion.

### Evolutionary education and understanding

Approximately 93% of participants agreed or strongly agreed the nutrition and dietetics field can benefit from incorporating outside fields of study. When asked how familiar participants were with the field of evolutionary medicine (full question in Supplementary Data S1), 50% were somewhat familiar and 43% were not at all familiar.

Of the participants who could recall, (*n* = 1710), 45% reported that no information about the theory of evolution was presented in their dietetic education. Roughly 25% reported the term was used but without detail and 28% said only some information was presented. Of those who reported any level of evolutionary education (*n* = 938; 55%), <42% (or 23% of total sample population) said some conversations on the theory of evolution incorporated aspects of health and disease, whereas 35% reported none of their discussions did.

Roughly 59% of participants reported they either agree or strongly agree they are knowledgeable about the theory of evolution, whereas 16% reported they disagree or strongly disagree, with 25% reporting unsure. Those who reported agree or strongly agree (*n* = 1101; 59%) then defined the theory of evolution in their own words. The four most common words used, excluding ‘Evolution’ and ‘Evolved,’ were: change, time, survival and adapt (Table 2).

**Table 2.** Participant’s evolution definition word/phrase frequency query

Word/Phrase included in definition	Students (*n* = 433)	Professionals (*n* = 494)	Total (*n* = 927)
Change	35%	39%	37%
Time	42%	37%	40%
Survival	32%	34%	33%
Adapt	32%	34%	33%
‘Natural Selection’	12%	10%	11%
‘Survival of the Fittest’	10%	9%	9%
‘Change Over Time’	6%	5%	6%

Word stems used in search:

Change: change, changed, changes, changing.

Time: time, times.

Survival: survival, survive, survived, survives, surviving.

Adapt: adapt, adaptability, adaptable, adaptation, adapted, adapting, adaptive, adapts.

### Evolutionary application

Approximately 58% of participants either agreed or strongly agreed an understanding of evolution can aid the nutrition and dietetics field, with only 11% selecting disagree or strongly disagree. Furthermore, 51% agreed or strongly agreed that incorporating an evolutionary perspective on health and disease would be beneficial in dietetics education, with only 12% reporting either disagree or strongly disagree. Lastly, 71% of participants either agreed or strongly agreed that, when trying to understand a disease, it is important to know both the ultimate (evolutionary) and proximate (direct) cause, with only 5% reporting either disagree or strongly disagree. Table 3 provides an overview of this information, including a summed mean for these three questions.

**Table 3.** Evolutionary medicine’s applicability to the field of nutrition and dietetics

	Strongly Agree (%)	Agree (%)	Neutral (%)	Disagree (%)	Strongly Disagree (%)	Mean	SD
An understanding of evolution can aid in the field of nutrition and dietetics.							
*n* = 1865	15	44	31	8	3	2.41	0.93
Incorporating an evolutionary perspective on health and disease would be beneficial in dietetics education.							
*n* = 1804	9	42	33	9	4	2.54	0.91
When trying to understand the pathology of a disease, it is equally important to understand both the ultimate (evolutionary) and proximate (direct) cause of the disease.							
*n* = 1868	18	53	24	4	1	2.17	0.81

						Summed mean	SD
						2.37	0.88

Participants were asked to select which areas within nutrition and dietetics would benefit from an evolutionary perspective (Supplementary Data S1). Both groups reported identical choices for the top five areas (in descending order): research, nutritional biochemistry, clinical nutrition, education and counseling.

### Clinical applicability

Over 46% of participants reported they were unlikely or very unlikely to provide an evolutionary explanation of a condition or a disease to a patient or a client, with 33% reporting neutrally. Participants were also given a clinical counseling scenario question, where 45% of participants reported they would be very unlikely or unlikely to *think* of an evolutionary-based explanation during a nutrition counseling session, with 29% reporting neutrally. Similarly, >53% of participants reported they would be very unlikely or unlikely to *provide* an evolutionary-based explanation to a patient or client, with >25% reporting neutrally.

### Participant responses by professionals versus students

We tested the hypothesis that professionals and students view evolutionary medicine differently. Students were more familiar with evolutionary medicine than professionals, with 52% of students reporting being somewhat familiar, compared to 46% of professionals reporting not at all (*P* < 0.0001). Additionally, where 1 indicates a lot of information and 3 indicates none, students reported receiving more education on the theory of evolution (students mean (±SD) 2.7 (±1.3) vs professionals 3.1 (±1.2), *P* < 0.0001). Where 1 indicates strongly agree and 5 strongly disagree, students agreed more than professionals that it is equally important to understand both the ultimate (evolutionary) and proximate (direct) cause of a disease (students 2.1 (±0.8) vs professionals 2.2 (±0.8), *P* < 0.0001) (Supplementary Data S2 for further analyses).

### Participant responses by US region

We tested our hypothesis that participant responses vary by US region. Where 1 indicates strongly agree and 5 strongly disagree, a potential association between the northeast region and the idea that an understanding of evolution can aid the nutrition and dietetics field was found (Northeast 2.2 (±0.8), South 2.5 (±1.0), Midwest 2.5 (±1.0), West 2.3 (±0.9), Total 2.4 (±0.9), *P* < 0.0006) (Supplementary Data S3).

### Participant responses by religious belief

Table 4 addresses the hypothesis that if a participant’s religious beliefs challenge the theory of evolution, then the participant will be less likely to support evolutionary medicine (Supplementary Data S4). Eight survey question responses were significantly (*P* < 0.0001) different between the three reported religious beliefs. Generally, participants who reported God made humans 10 000 years ago were less supportive of evolutionary application compared to other groups.

**Table 4.** Differences in response by participant’s religious belief

	Evolution without god guidance *n* = 600	God-guided evolution *n* = 820	God made humans 10 000 yrs. Ago *n* = 422	Total *n* = 1842	*P*-value*
How familiar are you with the field of evolutionary medicine, also known as Darwinian or ancestral medicine?					
Very familiar	9.20%	5.50%	4.70%	6.50%	<0.0001
Somewhat	51.70%	52.60%	43.60%	50.20%	
Not at all	39.20%	42.00%	51.70%	43.30%	
I am knowledgeable of the theory of evolution.					
Mean (SD)	2.2 (±0.9)	2.6 (±1.0)	2.6 (±0.9)	2.5 (±1.0)	<0.0001
When trying to understand the pathology of a disease, it is equally important to understand both the ultimate (evolutionary) and proximate (direct) cause of the disease.					
Mean and SD	2.0 (±0.7)	2.1 (±0.7)	2.5 (±0.9)	2.2 (±0.8)	<0.0001
An understanding of evolution can aid in the field of nutrition and dietetics.					
Mean and SD	2.0 (±0.8)	2.3 (±0.8)	3.2 (±1.0)	2.4 (±0.9)	<0.0001
Incorporating an evolutionary perspective on health and disease would be beneficial in dietetics education.					
Mean and SD	2.3 (±0.8)	2.4 (±0.8)	3.3 (±1.0)	2.5 (±0.9)	<0.0001
How likely are you to provide an evolutionary explanation of a condition or a disease to a patient or client?					
Mean and SD	3.1 (±1.0)	3.3 (±1.0)	3.9 (±1.0)	3.4 (±1.1)	<0.0001
Scenario: How likely would you be to think of an evolutionary-based explanation?					
Mean and SD	3.2 (±1.2)	3.2 (±1.2)	3.6 (±1.2)	3.3 (±1.2)	<0.0001
Scenario: How likely would you be to provide an evolutionary-based explanation?					
Mean and SD	3.4 (±1.1)	3.4 (±1.1)	3.9 (±1.1)	3.5 (±1.1)	<0.0001

*P-values from Fisher's exact test. For ease of presentation, some questions presented as mean (±SD) of Likert scale (1-strongly agree, 5-strongly disagree), but null hypothesis that categorical question answer was not associated with participant's religious belief still tested by Fisher's exact test.

### Question responses by participant’s reported evolutionary knowledge

Table 5 addresses the final hypothesis that self-reported evolutionary knowledge is associated with appreciation of evolutionary medicine (Supplementary Data S5). Six survey questions responses were significantly (*P* < 0.0001) different between reported evolutionary knowledge. As reported evolutionary knowledge increased, support for evolutionary application followed.

**Table 5.** Difference in response by participant’s prior evolutionary knowledge

	Strongly agree	Agree	Neutral	Disagree	Strongly disagree	Total	*P*-value*
	*n* = 221	*n* = 880	*n* = 472	*n* = 246	*n* = 49	*n* = 1868	
How much information about the theory of evolution has been presented to you in your program's coursework?							
Mean and SD	2.7 (±1.4)	2.8 (±1.3)	2.9 (±1.2)	3.2 (±1.1)	3.7 (±0.6)	2.9 (±1.3)	<0.0001
The field of nutrition and dietetics can benefit from incorporating outside fields of study.							
Mean and SD	1.4 (±0.6)	1.6 (±0.7)	1.6 (±0.7)	1.6 (±0.6)	1.6 (±0.8)	1.6 (±0.7)	<0.0001
When trying to understand the pathology of a disease, it is equally important to understand both the ultimate (evolutionary) and proximate (direct) cause of the disease.							
Mean and SD	1.9 (±0.9)	2.2 (±0.8)	2.3 (±0.8)	2.3 (±0.8)	2.6 (±0.8)	2.2 (±0.8)	<0.0001
An understanding of evolution can aid in the field of nutrition and dietetics.							
Mean and SD	1.9 (±0.9)	2.4 (±1.0)	2.5 (±0.8)	2.6 (±0.8)	2.7 (±0.9)	2.4 (±0.9)	<0.0001
Incorporating an evolutionary perspective on health and disease would be beneficial in dietetics education.							
Mean and SD	2.2 (±1.0)	2.5 (±0.9)	2.7 (±0.8)	2.7 (±0.8)	3.0 (±0.9)	2.5 (±0.9)	<0.0001
How likely are you to provide an evolutionary explanation of a condition or a disease to a patient or client?							
Mean and SD	2.9 (±1.2)	3.3 (±1.1)	3.5 (±1.0)	3.7 (±0.9)	3.9 (±1.0)	3.4 (±1.1)	<0.0001

*P-values from Fisher's exact test. For ease of presentation, questions presented as mean (±SD) of Likert scale (1- strongly agree, 5- strongly disagree), but null hypothesis that categorical question answer was not associated with participant's prior evolutionary knowledge still tested by Fisher's exact test.

## DISCUSSION

To our knowledge, this is the first study examining views on evolutionary medicine within the nutrition and dietetics field. This study produced several key findings: (i) it is unlikely to find structured education on evolutionary medicine within dietetic programs; (ii) participants were supportive of the notion that an understanding of evolution would benefit the field itself and dietetic education; (iii) though the majority of participants reported they were knowledgeable on the theory of evolution, their definitions of evolution lacked key components of biological evolution; (iv) it is unlikely an evolutionary perspective will be incorporated in clinical counseling situations; (v) current students likely receive more evolutionary education relative to what professionals received in the past; (vi) the Northeast US region may be more supportive of the idea that evolution can aid the nutrition and dietetics field; (vii) religious belief is associated with level of support for evolutionary medicine within the field; and (viii) though an understanding of evolution is not needed to support incorporating an evolutionary perspective, it does co-occur with higher levels of support.

Our dropout rate of 14% is higher than what is expected (10%) for online surveys [36]. This could be caused by the controversial nature of the theory of evolution [29, 30], or a possible aversion to evolution. Measures were taken to create a generalizable sample by including every possible email contact and keeping the survey title neutral towards the topic of evolution. Given the large sample size (*n* = 2039), relatively equal regional distribution, and similar gender percentages compared to available data, this suggests the sample population provides a strong reference for the field today.

Roughly 58% of US adults believe humans evolved over time, with or without guidance from God, and 38% believe God created humans in their present form [28]. Since scientists tend to support evolution more than the general US population [37], the scientific background of nutrition and dietetics could explain why roughly 77% of participants believe humans evolved over time, with or without guidance from God. Participant responses to religious beliefs, with and without dropouts defined as those believing God made humans 10 000 years ago, are significantly different from the Gallup 2018 results [28], suggesting participants support evolution more than the general US population. However, reclassifying the dropouts brings the responses closer to the 2018 Gallup population, as shown with the change in the X^2^ statistic (401.68 vs 252.58). Further, a higher number of participants (49%) believe Charles Darwin’s theory of natural selection is well-supported by evidence compared to the general population (35%) [40]. Similarly, fewer participants (28%) compared to the general population (35%) believe the theory of natural selection is just one of many theories and is not well-supported by evidence [38].

Interestingly, 79% of the US population reported they are very or somewhat familiar with an evolutionary explanation about the origin and development of life on earth [38]. This might explain why 59% of participants reported agreeing or strongly agreeing they are knowledgeable on the theory of evolution, even though 70% reported no information about the theory of evolution was presented in their dietetic education or the term was used without meaning.

Only 23% of total participants reported some of their discussions on evolution incorporated aspects of health and disease, suggesting only a small subset of students may be learning about evolution’s impact on health and disease. However, participant definitions suggest only a superficial evolutionary understanding, with the two most reported words, ‘change’ and ‘time’, being generic evolutionary terms. Further, biological evolution key points (e.g. fitness, genetic variation and inheritance) were rarely presented and only 11% of the participants used ‘natural selection’ in their definitions. Also, common misconceptions of evolution and natural selection were present in the reported definitions: teleology, anthropomorphism and evolution in response to need [39]. Given these results and the lack of evolutionary education required by the ACEND, these data support the hypothesis that nutrition and dietetics students are not learning about the theory of evolution and its application to health and disease.

Surprisingly, 50% of participants reported they are somewhat familiar with evolutionary medicine. This could be attributed to the popularity of the controversial Paleo Diet [40], which may be discussed in nutrition and dietetics coursework. Still, 43% reported they were not at all familiar with evolutionary medicine, while most participants were supportive of an evolutionary perspective aiding the nutrition and dietetics field, and specifically in dietetic education. Furthermore, the summed mean calculated in Table 3 shows participants are overall supportive (2.37) of incorporating an evolutionary perspective to the nutrition and dietetics field. Additionally, 71% of participants support the notion that it is equally important to understand both the ultimate (evolutionary) and proximate (direct) cause of a disease. These results provide evidence that rejects our hypothesis, suggesting nutrition and dietetics professionals and students appreciate evolutionary medicine.

It has been argued that evolutionary biology should be a basic science for medical education [41], and nutrition and dietetics falls within the medical field. Importantly, when students learn a new concept they will often ask ‘Why?’ to acquire a deeper understanding. As suggested for medical students [18], being able to answer a nutrition and dietetics students’ ‘Why’ questions with an evolutionary-based response will supply students with the methodology needed to understand the proximate causes of today’s diseases. Additionally, it has been reported that an evolutionary perspective enhances the understanding of human nutritional requirements [4]. Collectively, given the above literature and our survey results, incorporating an evolutionary perspective in dietetics education would likely produce beneficial results.

The clinical applicability of an evolutionary perspective in medicine has been presented [42, 43]; however, negative criticisms also exist [44]. Participants reported mixed support of evolutionary medicine’s clinical applicably throughout the survey. Both clinical nutrition and counseling were ranked in the top five areas that would benefit from an evolutionary perspective, while questions specific to clinical nutrition and counseling revealed low support. This may suggest participant support of including an evolutionary medicine perspective in clinical research, but outside of a counseling setting. Since a nutrition and dietetics professional’s role is often bed-side and very personal, specifically with nutritional counseling, avoidance of the controversial topic of evolution in this religious and culturally sensitive setting may explain why participants were less supportive of using an evolutionary approach in counseling.

Interestingly, students reported being significantly more familiar with evolutionary medicine and having had more evolutionary education presented to them compared to professionals who could recall. This could be attributed to the average 19-year age difference between student and professional participants or reflect the reported growth of evolutionary medicine [45], suggesting future nutrition and dietetics professionals might be more likely to incorporate an evolutionary perspective than current professionals. Furthermore, this could explain why students were significantly more in favor of understanding both the ultimate and proximate causes of disease.

We hypothesized that US region may be associated with evolutionary support since, for example, it has been shown that religiosity in the southern region rather than education best predicts a student’s understanding of evolution [46]. US region appeared to minimally affect results, as all regions were supportive of the idea that an understanding of evolution can aid the nutrition and dietetics field. However, a potential association between the Northeast and support of evolutionary medicine compared to other US regions was found.

Since religious beliefs can challenge acceptance of evolution, we hypothesized that if a participant’s religious belief challenged the theory of evolution, then it would reduce evolutionary support. For many of the questions presented in Table 4, the mean response decreased (showing less support for evolutionary application) by each group, as predicted by the hypothesis. Overall, the mean was more supportive for participants who believe in evolution without guidance by God, lower for those who believe in God-guided evolution, and lowest for those who believe God made humans in their present form. These results support this hypothesis and the notion that it might be unfavorable to incorporate this approach during a counseling session, where cultural background is an important factor.

Lastly, we tested if evolutionary knowledge was needed to appreciate this interdisciplinary approach. As predicted, those who strongly agreed they are knowledgeable of the theory of evolution also reported more evolutionary education during their dietetics program. Overall, as reported evolutionary knowledge increased, support for evolutionary application followed. However, regardless of reported evolutionary knowledge, all levels were in favor (neutral or supportive) of evolutionary medicine. These data do not support the hypothesis that evolutionary knowledge is associated with appreciation of evolutionary medicine.

Study strengths include: (i) large sample size, (ii) concealing the potential controversial survey topic until consent was obtained, (iii) mixed sample population of students and professionals and (iv) equal US region distribution. Since we used a mix of students and professionals, this allows us to comment on where the field may be headed regarding evolutionary medicine in the future. Additionally, redundant questions were used for various themes within the survey (e.g. clinical application and evolutionary understanding). Limitations include: (i) not all dietetic professionals are AND members and thus did not have the possibility to participate, (ii) the sample is a convenience non-probability sample, (iii) a response rate is impossible to calculate and (iv) an interviewer was not used, limiting the data collection. Additionally, it is possible participants could have produced a social desirability bias and answered questions based on survey content rather than personal views [47]. Nonresponse bias was minimized by distributing the survey to every contact from eatright.org, pretesting survey material, allowing ample time for survey completion (≥30 days), sending out follow-up emails to encourage participation, providing incentive, and by excluding the word ‘Evolution’ in the survey title. It is also important to note participants who were supportive of interdisciplinary approaches and/or evolution may have been more likely to complete the full survey, thus skewing the results in a positive manor.

Future research could study how an evolutionary approach might be best incorporated into nutrition and dietetics education. Fotunately, much information has already been written about incorporating evolutionary biology into medical education [16, 18, 41], and could be applied to content in nutrition and dietetics programs. Additionally, determining which evolutionary medicine content is most relevant for the field of nutrition and dietetics is still needed. Lastly, future studies could clarify the mixed data presented here and determine the application of evolutionary medicine into a clinical nutrition and counseling setting.

In conclusion, our study suggests there is support within the field to incorporate evolutionary medicine into nutrition and dietetics. Since nutrition and dietetics professionals hold such a crucial role in the medical field, learning about this keystone of biological sciences and its applications to health and disease today would be advantageous. Specifically, as both students and professionals reported evolutionary medicine would be beneficial in dietetics education, perhaps future educational guidelines could incorporate this interdisciplinary approach. Literature examining the intersection of evolutionary medicine within the nutrition and dietetics field has been non-existent until now. In an era of growing chronic disease and rising healthcare costs, all viable approaches should be considered.

## Funding

This research did not receive any specific grant from funding agencies in the public, commercial or not-for-profit sectors.
